# MiR-93 regulates vascular smooth muscle cell proliferation, and neointimal formation through targeting Mfn2

**DOI:** 10.7150/ijbs.36995

**Published:** 2019-09-07

**Authors:** Shengdong Feng, Lu Gao, Dianhong Zhang, Xinyu Tian, Lingyao Kong, Huiting Shi, Leiming Wu, Zhen Huang, Binbin Du, Cui Liang, Yanzhou Zhang, Rui Yao

**Affiliations:** Department of Cardiology, the First Affiliated Hospital of Zhengzhou University, Zhengzhou, China

**Keywords:** miR-93, vascular smooth muscle cells, proliferation, migration, Mfn2

## Abstract

**Background/Aims**: Vascular smooth muscle cell (VSMC) hyperplasia plays important roles in the pathogenesis of many vascular diseases, such as atherosclerosis and restenosis. Many microRNAs (miRs) have recently been reported to regulate the proliferation and migration of VSMC. In the current study, we aimed to explore the function of miR-93 in VSMCs and its molecular mechanism.

**Methods**: First, qRT-PCR and immunofluorescence assays were performed to determine miR-93 expression in rat VSMCs following carotid artery injury in vivo and platelet-derived growth factor-BB (PDGF-BB) stimulation in vitro. Next, the biological role of miR-93 in rat VSMC proliferation and migration was examined in vivo and vitro. EdU incorporation assay and MTT assay for measuring cell proliferation, Transwell cell invasion assay and Cell scratch wound assay for measuring cell migration. Then, the targets of miR-93 were identified. Finally, the expression levels of proteins in the Raf-ERK1/2 pathway were measured by western blot.

**Results**: MiR-93 was upregulated in rat VSMCs following carotid artery injury in vivo. Similar results were observed in ex vivo cultured VSMCs after PDGF-BB treatment. MiR-93 inhibition suppressed neointimal formation after carotid artery injury. Moreover, our results demonstrated that a miR-93 inhibitor suppressed the PDGF-BB induced proliferation and migration of in VSMC. This inhibitor also decreased the expression levels of MMP2 and cyclin D1. Mechanistically, we discovered that mitofusin 2(Mfn2) is a direct target of miR-93. Furthermore, an analysis of the signaling events revealed that miR-93-mediated VSMC proliferation and migration occurred via the Raf-ERK1/2 pathway.

**Conclusions**: Our findings suggest that miR-93 promotes VSMCs proliferation and migration by targeting Mfn2. MiR-93 may be a new target for treating in-stent restenosis.

## Introduction

Cardiovascular disease is one of the most important diseases that threaten human life. Many studies have confirmed that abnormal vascular smooth muscle proliferation plays a key role in cardiovascular diseases, such as atherosclerosis and atherosclerotic restenosis 1-3. In response to injury and other stimuli, the expression levels of contractile proteins are decreased, and the expression levels of proliferating proteins are increased in vascular smooth muscle cells (VSMCs); these changes lead to increased cell proliferation, and it plays a role in the pathogenesis of various cardiovascular diseases, including vascular restenosis after angioplasty 4, 5. However, the molecular mechanism of vascular restenosis after angioplasty is not fully understood. Thus, a better understanding of these regulatory mechanisms of vascular restenosis might lead to novel strategies for suppressing cardiovascular disease.

MicroRNAs (miRNAs) are a class of small non-coding RNAs that bind to the 3'untranslated region (3'UTR) of specific mRNAs to cause the degradation of target gene mRNA. Currently, more than 2,000 miRNAs have been found in the human genome. Studies have shown that miRNAs play roles in many diseases 6, 7. Recently, multiple studies 8-10 suggest that miRNAs, such as miR-132^11^, miR-22^12^, miR-379^13^, miR-124^14^, miR-214^15^, miR-221/miR-222^16^, miR-146^17^, and miR-222^18^, play pivotal roles in VSMC proliferation and migration.

MiR-93 belongs to the miR-17 microRNA cluster and is closely related to the early evolution of the vertebrate lineage 19. Previous studies have demonstrated that miR-93 is involved in various disease processes, including cancer 20-22, myocardial ischemia-reperfusion 23, and spinal cord neurons damages 24. However, whether miR-93 is involved in VSMC proliferation is not fully understood. In the present study, we identified miR-93 as a novel regulator of VSMC proliferation, differentiation and arterial injury-induced neointimal hyperplasia. We reported that miR-93 promotes VSMC proliferation and migration by targeting mitofusin- 2 (Mfn2), which might be a new therapeutic target for neointimal hyperplasia.

## Materials and Methods

### Animal experiments

All of the animal protocols were approved by the Animal Care and Use Committee of the First Affiliated Hospital of Zhengzhou University. The animal experiments were performed in accordance with the National Institutes of Health Guidelines for the Care and Use of Laboratory Animals (NIH Publication No. 80-23, revised in 1996). Adult Sprague-Dawley male rats (250 g-300 g) were used for the carotid artery balloon injury model. Rats were anesthetized intraperitoneally with 10% chloral hydrate (3 ml / kg). Tissues were separated from the skin, and the left carotid artery was exposed. The carotid artery and the internal carotid artery were clamped with a vascular clip. Then, a microscope was used, and a small opening was cut in the external carotid artery. A balloon catheter with a diameter of 2.0 mm was inserted into the proximal end (Pioneer, Shanghai, China). The balloon was inflated with air and pulled through the artery ten times. Then, the balloon was deflated, the external carotid artery was ligated, the internal carotid artery clip was opened, and the wound was sutured. For the sham injury group, the carotid artery was exposed, and there was no damage to the blood vessels. The antagomir-93 group and the control group were injected with 200μl (0.5 nmol/μl) of antagomir-93 or antagomir-93 control every 3 days. The injections began from the week before the operation and continued until 14 days after surgery. The left common carotid artery was fixed in paraffin and sliced, we select the middle of the artery for H & E staining or tissue immunofluorescence. The animals were divided into a carotid artery balloon injury group treated with the miR-93 antagomir control and a carotid artery balloon injury group treated with the miR-93 antagomir.

### Cell culture and treatment

Rat VSMCs were extracted from the aortas of six-week-old Sprague-Dawley male rats by using tissue adhesion methods. Blood vessels were cut out, and the fat, connective tissue and fibrous membrane were peeled off. The tissue was then cut into small pieces and transferred to a cell culture flask for overnight incubation with DMEM-F12 supplemented with 10% fetal bovine serum (FBS) in a humidified 5% CO_2_ atmosphere at 37 °C. Primary VSMCs between the 3rd and 6th passages were used for experiments. Human 293T cells were maintained in DMEM supplemented with 10% FBS in a humidified 5% CO_2_ atmosphere at 37 °C.

MiR-93 mimics, miR-93 inhibitors, antagomiR-93 and matched controls were purchased from RiboBio (Guangzhou, China). Mfn2 siRNA, Mfn2 pcDNA and matched controls were purchased from RiboBio (Guangzhou, China). Lipofectamine 2000 (Invitrogen, Waltham, MA, USA) was used to perform miRNA transfections according to the manufacturer's instructions. PDGF-BB (Sigma, St. Louis, MO, USA) was used in time and concentration gradient experiments to determine the final intervention dose.

### Immunofluorescent detection of miRNA-93 in rat aortic SMCs

The expression and localization of miR-93 were determined according to a previously described immunofluorescence method 25. Rat VSMCs were extracted from the aortas of six-week-old Sprague-Dawley male rats by using tissue adhesion methods. After subculture, we selected vascular smooth muscle cells from the third to the fifth generation for immunofluorescent detection. The probes and interfering probes for miR-93 were labeled with the MEGAscript ™ T7 Transcription Kit (Invitrogen) in vitro. VSMCs were placed in a buffer solution (99.5 ml of 0.1 mol citrate buffer and 0.5ml of Triton X-100) at room temperature for 10 min. After the VSMCs were dried, they were placed in a low-dose pepsin working solution (1 ml 3% citric acid and 2 drops of pepsin solution) for 20 min at 37 °. The cells were then washed three times with PBS washed and once with, 0.2 × SSC (1.753g of NaCl and 0.882g of trisodium citrate in sterilized water to a volume of 1000 ml) for 3 min at room temperature. Afte the excess liquid on the tissue was dried, prehybridized working solution (one drop of salmon sperm DNA; the final concentration of the salmon sperm DNA was 100 µg/ml) was added to cover the tissue. Next, the samples were covered with in situ hybrid coverslips and incubated for 2 h at 42 ℃ in a wet box. Then, the coverslips were removed, and the samples were washed with 0.2 × SSC at room temperature three times; each wash was for 5 min. After removing the excess liquid from the tissue, the hybridization solution was added, (the probe was diluted with a super hybridization solution containing 100 µg/ml salmon sperm DNA). The samples were washed with 2 × SSC for 3min at 37 ℃, three times with 0.2 × SSC for 5min at 37 ℃ and three times with PBST for 5min at 37 ℃. Finally, the samples were incubated with FITC (Invitrogen) and DAPI solutions (Thermo Fisher, Waltham, MA, USA), washed with PBST, observed under a fluorescence microscope and photographed.

### Western blotting

Cells or tissues were homogenized in ice-cold suspension buffer (RIPA Lysis Buffer) supplemented with a proteinase inhibitor cocktail (Sigma-Aldrich). Briefly, the protein concentrations were determined using a BCA protein assay kit (Thermo Fisher). Equal amounts of protein were fractionated on SDS polyacrylamide gels, followed by immunoblotting with the following primary antibodies: anti-cyclin D1 (1:1,000, Abclonal,Wuhan,China), anti-MMP2 (1:1,000, Abclonal), anti-Mfn2 (1:1,000, Abclonal), anti-Erk1/2 (1:1,000, Abclonal), anti-phospho-Erk1/2(1:1,000, Abclonal), anti-Raf (1:1,000, Abclonal), anti-phospho-Raf (1:1,000, Abclonal), anti-β-actin (1:100, Abclonal), and anti-GAPDH (1:1,000, Proteintech). Membranes were then incubated with a peroxidase-conjugated secondary antibody, and specific bands were detected with a Bio-Rad (Hercules, CA) imaging system.

### RNA isolation and quantitative real-time PCR

Total RNA was extracted from cells with TRIzol reagent (D9108A; Takara Bio). RNA was reverse-transcribed using an RNA PCR kit (RR036A; Takara Bio). Quantitative real-time PCR amplification was performed with an ABI PRISM 7900 Sequence Detector system (Applied Biosystem, Foster City, CA), according to the manufacturer's instructions.

### EdU incorporation assay

EdU incorporation assays were performed using Cell-Light EdU Apollo®567 In Vitro Imaging Kits (RiboBio). The logarithmic growth was determined for VSMCs seeded in 96-well plates at 4×10^3^ cells per well. After 24 h of the indicated treatment, each well was incubated with 100μl of 50μM EdU medium for 2 h. The cells were fixed in PBS containing 4% paraformaldehyde for 30 min. Then, 2 mg/ml glycine was added for 5 min. After washing with PBS, the cells were incubated with 1×Apollo staining solution for 30 min. The staining solution was discarded, and the cells were washed with PBS containing 0.5% TritonX-100 for 10 min. After washing again with PBS, 1×Hoechest 33342 was added for a 30 min incubation at room temperature. After washing with PBS, the positive cells were observed by fluorescence microscopy.

### Cell viability assay

Cell viability was measured by MTT assay. The logarithmic growth was determined for VSMCs seeded in 96-well plates at 5×10^3^ cells per well. After 24 h of the indicated treatment, 20 μl of MTT solution (5 mg/ml, Sigma) was added to each well, incubated for 4 h and discarded. Next, 150 µl of dimethyl sulfoxide (DMSO, Sigma) was added to each well. The plates were shaken at a low speed for 10 min, so that the crystals fully dissolved. The absorbance values for each well were measured at OD490 nm. A blank well (medium, MTT, DMSO) was included for comparison.

### Transwell cell invasion assay

After 24 h of the indicated treatment, the cells were digested and resuspended in serum-free medium. A total of 100μl of cell suspension (5 x 10^4^) was added to the upper chambers of a Transwell culture plate. To the bottom chambers, 500 μl of medium containing 10% FBS was added. The plate was incubated at 37 °C and 5% CO2 for 24 h. The cells on the upper surface of the polycarbonate films were gently removed with wet cotton swabs. The polycarbonate films were carefully removed from the upper chambers, and the cells were fixed in pre-cooled methanol for 30 min. Then, the cells were stained with hematoxylin for 1 min, washed 3 times with PBS and observed under a microscope.

### Cell scratch wound assay

VSMCs were seeded in a six-well plate and transfected and treated. After 24 h, a straight line was drawn across the plate with a pipette tip, and 2% FBS medium was added. Images of the scratched cells were taken after 0 h, 12 h and 24 h using a microscope. The migration ability by the cells was analyzed according to the healed area of the scratch.

### Immunofluorescence

VSMCs were fixed in 4% paraformaldehyde and washed three times with PBS. Then, the cells were permeabilized with 0.1% Triton for 30 min. Next, the cells were washed 3 times with PBS and blocked with 1% BSA for 1 h. Then cells were incubated with primary antibodies (α-SMA and Mfn2) diluted 1:100 in 1% BSA overnight at 4 °C. After washing with PBST 3 times, the following fluorescent secondary antibodies (Thermo Fisher) were added: Alexa Fluor® 488 for Mfn2 and Alexa Fluor® 594 for α-SMA. After incubation for 1 h in 37 °C, the cells were washed 3times with PBST. Then, the cells were incubated with DAPI solution for 5 min in the dark, wash with PBST and examined under a fluorescence microscope. Tissue sections were dewaxed with xylene and ethanol, and the antigen was then repaired with 0.1 M citrate buffer. The primary antibodies included α-SMA and Mfn2, and the secondary antibodies were Alexa Fluor® 488 for α-SMA and Alexa Fluor® 594 for Mfn2.

### Luciferase assay

The PsiCHECK-2 vector (Promega, Beijing, China) containing both firefly and renilla luciferase genes was used. The 3'UTR of Mfn2 was cleaved from the genome by PCR and then linked to the linearized PsiCHECK-2 vector by ligating the 3'UTR of the construct to the PsiCHECK-2 vector containing the Mfn2 3'UTR. The vector was co-transfected with miR-93 mimic, miR-93 inhibitor or miR-93 control. After incubation for 48 h, Firefly and Renilla luciferase activities were detected with the Dual-Glo Luciferase Assay system (Promega).

### Statistical analysis

GraphPad Prism version 6 (GraphPad Prism version 6.0, La Jolla, CA, USA) was used to perform all statistical analyses. All data are expressed as the mean ± SE. Significant differences between groups were determined by t-test or one-way ANOVA followed by Bonferroni's multiple comparison tests. A P-value < 0.05 was considered statistically significant.

## Results

### MiR-93 was upregulated in proliferating VSMCs both in vivo and in vitro

**Figure 1:**
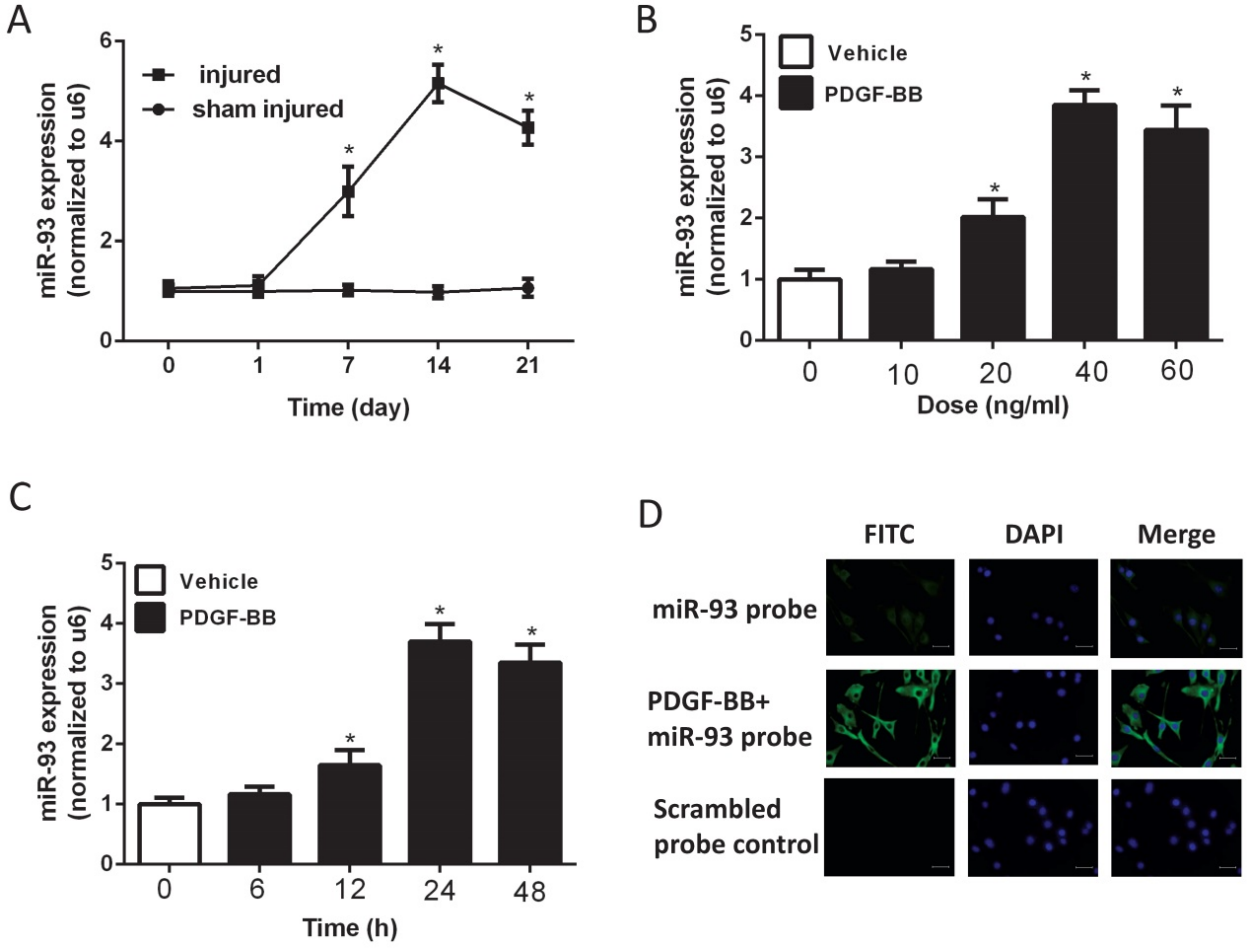
** MiR-93 is upregulated in proliferating vascular smooth muscle cells (VSMCs) both *in vivo* and *in vitro*.** (A) MiR-93 expression in the carotid arteries from rats at 1d, 7d,14d and 21d after balloon injury (*P<0.05 versus day 0, n=6); (B) platelet-derived growth factor-BB (PDGF-BB) caused a dose-dependent increase in miR-93 expression levels in VSMCs after treatment for 24 h, as demonstrated by qRT-PCR. (*P<0.05 versus 0 ng/ml, n=4); (C) PDGF-BB (40 ng/ml) caused a time-dependent increase in miR-93 expression levels in VSMCs as demonstrated by qRT-PCR. (*P<0.05 versus 0 h, n=4). (D) Immunofluorescence was performed to detect miR-93 (green) in VSMCs; blue indicates DAPI-stained nuclei (scale bar, 25 µm).

To investigate the expression level of miR-93 in VSMC proliferation, we used a catheterization-induced rat vascular injury model and determined the expression levels of miR-93 at different time points after the injury via qRT-PCR. MiR-93 levels were significantly higher 7 and 14 days after vascular injury than after sham injury. Interestingly, miR-93 levels were partially restored 21 days after injury (Fig. 1A). To further confirm the expression of miR-93 in proliferating VSMCs, qRT-PCR was performed in rat aortic SMCs stimulated with different doses of PDGF-BB. As shown in Fig. 1B, PDGF-BB stimulation significantly upregulated miR-93 expression in a dose-dependent manner, with a peak value of 40 ng/ml. In addition, PDGF-BB (40 ng/ml) stimulation significantly upregulated the miR-93 expression at different time points, with a peak value at 24 h (Fig. 1C). Immunofluorescent staining indicated that miR-93 is primarily expressed in the cytoplasm of quiescent SMCs and is upregulated in proliferating cells (Fig. 1D). Taken together, these findings indicate a positive relationship between miR-93 expression and VSMC proliferation.

### A miR-93 inhibitor prevented neointimal formation after carotid artery balloon injury in rats

**Figure 2:**
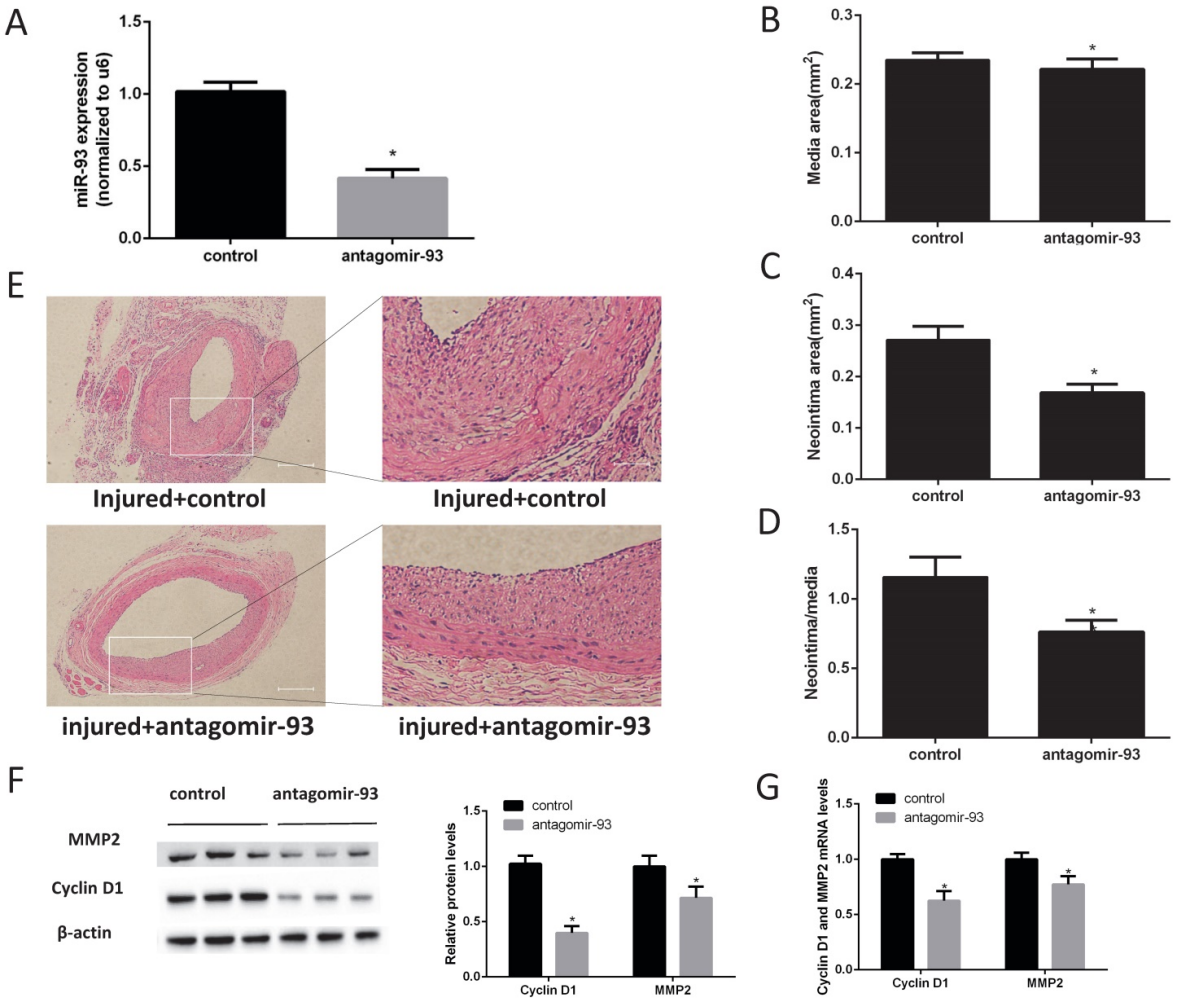
**MiR-93 inhibitor prevents neointimal formation after carotid artery balloon injury in rats.** Angioplasty followed by infection with antagomir-93 or the antagomir-93 control was performed on the rat left carotid arteries of rats. Rats were sacrificed 14 days after injury. (A) Analysis of miR-93 mRNA expression levels in rat left carotid arteries by qRT-PCR (*P<0.05 versus control, n=6) (B) Media areas of rat carotid cross-sections (*P>0.05 versus control, n=6). (C) Neointima areas of rat carotid cross-sections (*P<0.05 versus control, n=6). (D) Neointima/media areas of rat carotid cross-sections (*P<0.05 versus control, n=6). (E) Representative H & E staining of injured carotid arteries (left images, scale bar, 200 µm; right images, scale bar, 50 µm). (F) Analysis of cyclin D1 and MMP2 protein expression levels by western blot (*P<0.05 versus control, n=6). (G) Analysis of cyclin D1 and MMP2 mRNA expression levels in injured left carotids by qRT-PCR (*P<0.05 versus control, n=6).

Given the above data, we hypothesized that a miR-93 inhibitor may prevent the progression of restenosis. Therefore, carotid artery balloon injury rats were injected with a locked nucleic acid-modified antagomir-93 (miR-93 inhibitor) or a scrambled miR control in their caudal vein. The miR-93 inhibitor can significantly reduce the expression of miR-93 in the carotid artery (Fig. 2A). Morphometric analysis showed no difference in the media sizes of the antagomir-93-treated and antagomir-93 control-treated carotids (Fig. 2B). Interestingly, the neointima and the neointima/media ratio were markedly attenuated by antagomir-93 treatment (Fig. 2C-D). We can see it intuitively in the tissue section that the miR-93 inhibitor significantly reduced neointimal thickness (Fig. 2E). Cyclin D1 and MMP2 are critical regulators VSMCs proliferation and migrations 26-28. Compared with the control group, the antagomir-93 group had significantly reduced protein expression levels of cyclin D1 and MMP2 (Fig. 2F). In addition, the mRNA levels of cyclin D1 and MMP2 were markedly attenuated by antagomir-93 treatment (Fig. 2G). These results clearly indicated that a miR-93 inhibitor could block the neointimal hyperplasia induced by catheterization injury.

### A miR-93 inhibitor prevented VSMC proliferation and migration

**Figure 3:**
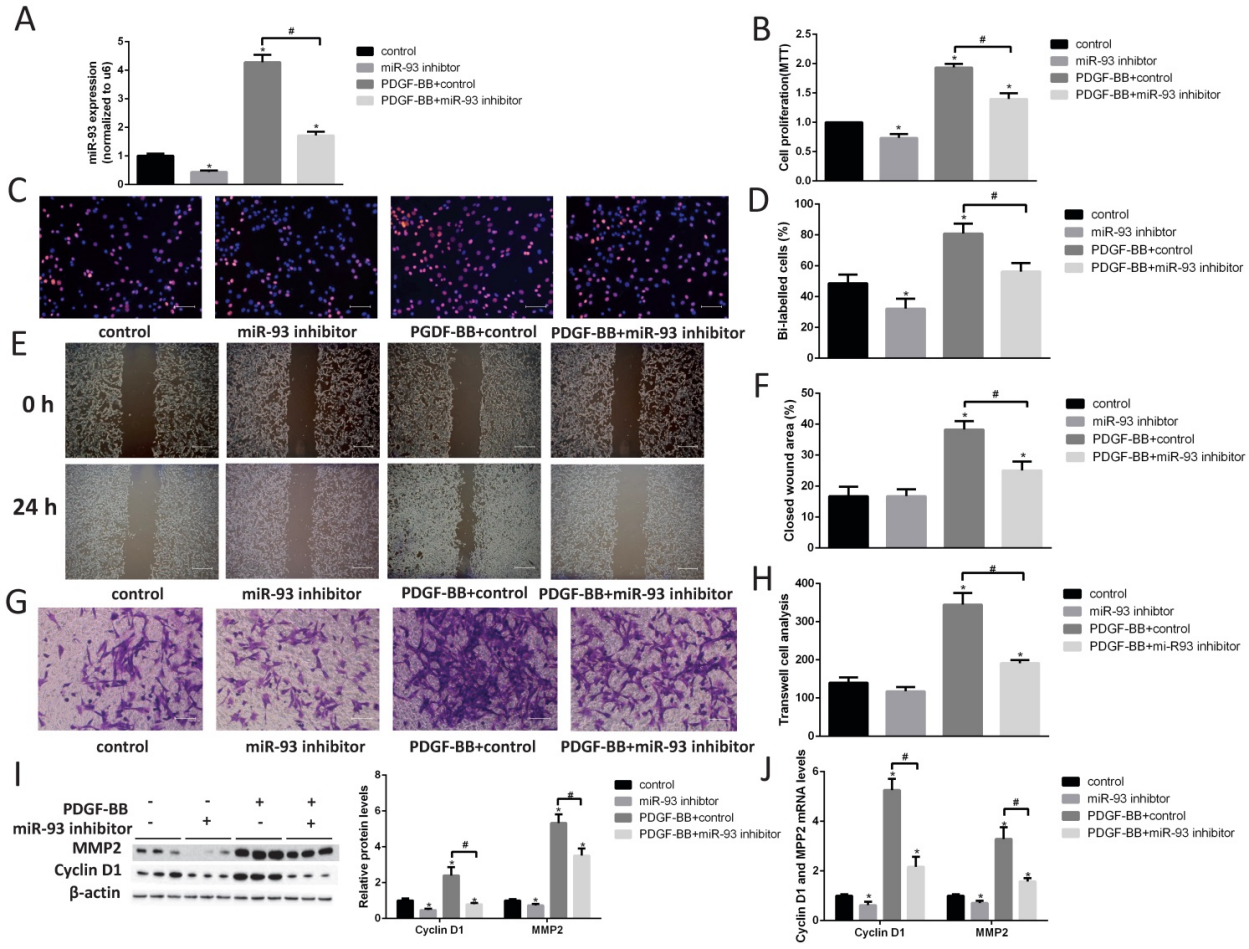
**MiR-93 inhibition prevents VSMC proliferation and migrations.** Rat VSMCs were transduced with a miR-93 inhibitor control (100 nmol) or miR-93 inhibitor (100 nmol). Serum-starved VSMCs were stimulated with or without PDGF-BB (40 ng/mL) for 24 h. (A) Analysis of miR-93 mRNA expression levels in rat VSMCs by qRT-PCR (*P<0.05 versus control, #P<0.05 compared between crossed lines, n=4). (B) The proliferation ability of VSMCs was measured by MTT assay (*P<0.05 versus control, #P<0.05 compared between crossed lines, n=4). (C) EdU incorporation in VSMCs; purple indicates the EdU-positive signal merged with nuclei stained with Hoechst 33342, and blue indicates nuclei (scale bar, 50 µm). (D) EdU-positive cells were quantified by Image-Pro Plus (*P<0.05 versus control, #P<0.05 compared between crossed lines, n=4). (E) VSMC migration ability was measured by scratch wound assays (scale bar, 100 µm). (F) Scratch wound assays were quantified by using ImageJ to measure the closed wound area (*P<0.05 versus control, #P<0.05 compared between crossed lines, n=4;). (G) VSMC migration ability was measured by Transwell assays (scale bar, 50 µm). (H) Migrated cells were quantified by Image-Pro Plus (*P<0.05 versus control, #P<0.05 compared between crossed lines, n=4). (I) Analysis of cyclin D1 and MMP2 protein expression levels by western blot (*P<0.05 versus control, #P<0.05 compared between crossed lines, n=4). (J) Analysis of cyclin D1 and MMP2 mRNA expression levels in rat VSMCs by qRT-PCR (*P<0.05 versus control, #P<0.05 compared between crossed lines, n=4).

Next, we transfected a miR-93 inhibitor into primary cultured VSMCs and examined cell proliferation by performing EdU and MTT assays. We first demonstrated that miR-93 inhibitor can significantly reduce the expression of miR-93 in VSMCs (Fig. 3A). The miR-93 inhibitor significantly attenuated VSMC proliferation both with and without PDGF-BB stimulation (Fig. 3B-D). Furthermore, we performed cell migration assays to determine whether miR-93 is also involved in VSMC migration. As shown in Fig. 3E-F, the wound healing area in VSMCs subjected to PDGF-BB stimulation induced was significantly reduced after transfection with the miR-93 inhibitor. Interestingly, the closed wound area was not significantly changed after transfection with the miR-93 inhibitor and without PDGF-BB stimulation. Transwell assays also confirmed that miR-93 inhibition could inhibit the VSMC migration induced by PDGF-BB (Fig. 3G-H). According to western blot analyses, the protein levels of cyclin D1 and MMP2 were significantly upregulated by PDGF-BB stimulation. However, transfection of the miR-93 inhibitor reversed these responses (Fig. 3I). We next performed qRT-PCR assays to determine the mRNA levels of cyclin D1 and MMP2, and we obtained similar results (Fig. 3J). These results suggest that miR-93 inhibition can alleviate PDGF-BB-induced VSMC proliferation and migration.

### Mfn2 was down regulated in proliferating VSMCs via a miR93-dependent pathway

**Figure 4:**
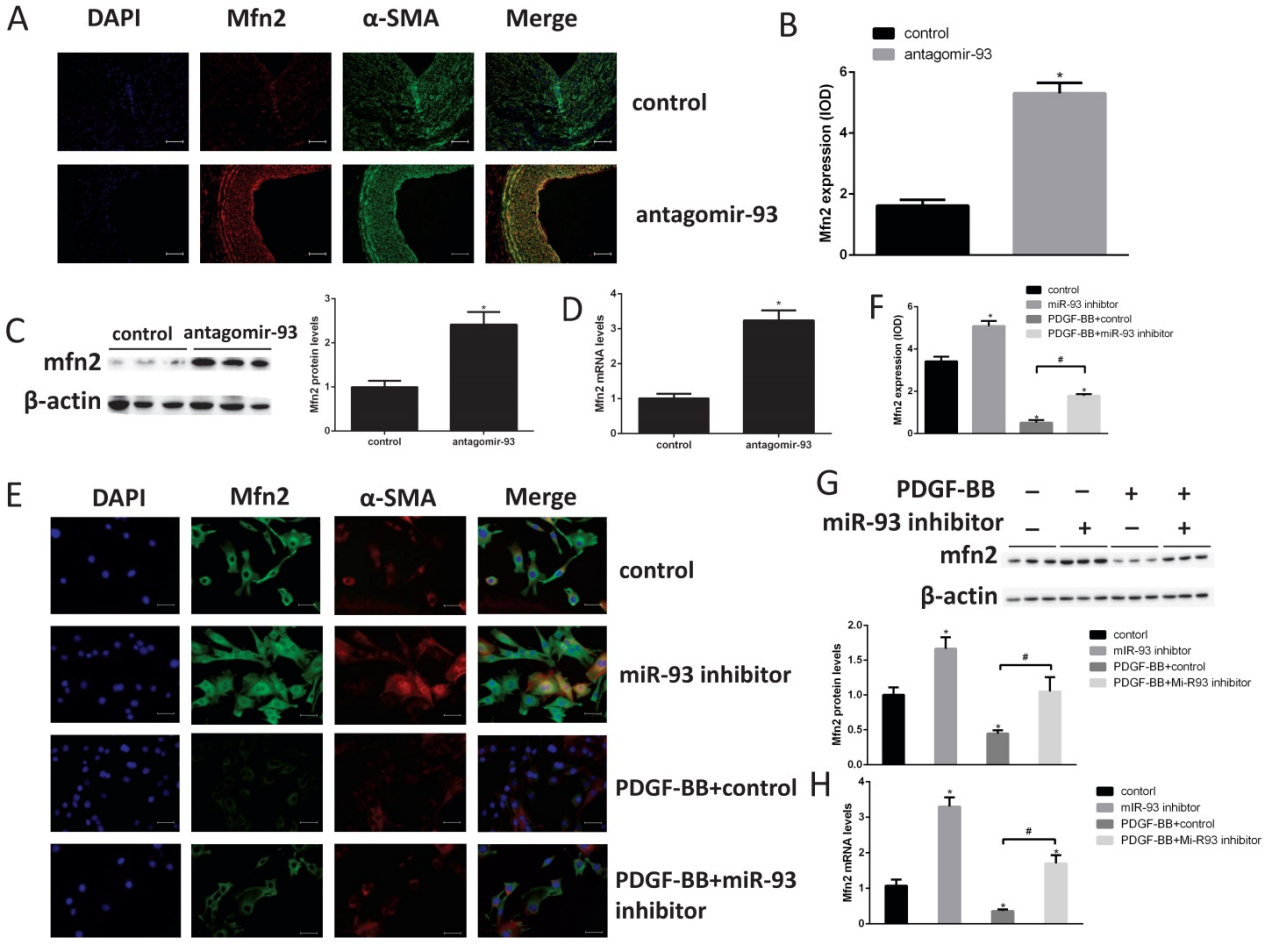
** Mfn2 is downregulated in proliferating VSMCs via a miR-93-dependent pathway.** (A) Immunofluorescence staining for Mfn2 was performed to determine Mfn2 protein expression in rat neointimas 14 days after injury. (B) The corresponding OD values are presented. (C) Analysis of Mfn2 protein expression levels in injured rat left carotids by western blot (*P<0.05 versus control, n=6). (D) Analysis of Mfn2 mRNA expression levels in injured rat left carotids by qRT-PCR (*P<0.05 versus control, n=6). (E) Mfn2 was detected in rat VSMCs in the indicated groups using cell immunofluorescence assays (scale bar, 25 µm). (F) The corresponding OD values are presented. (G) Analysis of Mfn2 protein expression levels in rat VSMCs by western blot (*P<0.05 versus control, #P<0.05 compare between crossed lines, n=4). (H) Analysis of Mfn2 mRNA expression levels by qRT-PCR, in rat VSMCs (*P<0.05 versus control, #P<0.05 compare between crossed lines, n=4).

To elucidate the molecular mechanism of miR-93 in regulating VSMC proliferation, we screened the predicted target candidates of miR-93 in silico to identify genes related to cell cycle regulation. Mfn2 is a dynamin-like protein involved in mitochondrial membrane rearrangement, and it plays a key role in maintaining mitochondrial network structure and normal mitochondrial metabolism 29. In addition, Mfn2 plays an important role in VSMC proliferation 30. According to the immunohistochemical analyses, we found the Mfn2 expression was markedly increased in neointima after transfection with antagomir-93 (Fig. 4A-B). This result was further confirmed by western blot and qRT-PCR assays as shown in Fig. 4C-D. Furthermore, cell immunofluorescence assays showed that the fluorescence intensity of Mfn2 was brighter when the miR-93 inhibitor was used both with and without PDGF-BB treatment (Fig. 4E-F). Finally, we verified these results by western blot and qRT-PCR assays as shown in Fig. 4G-H. The miR-93 inhibitor upregulated the expression of Mfn2 regardless of PDGF-BB treatment. These results suggested that the expression of Mfn2 was downregulated in proliferating VSMCs via a miR-93-dependent pathway.

### Identification of Mfn2 as a target gene of miR93

**Figure 5:**
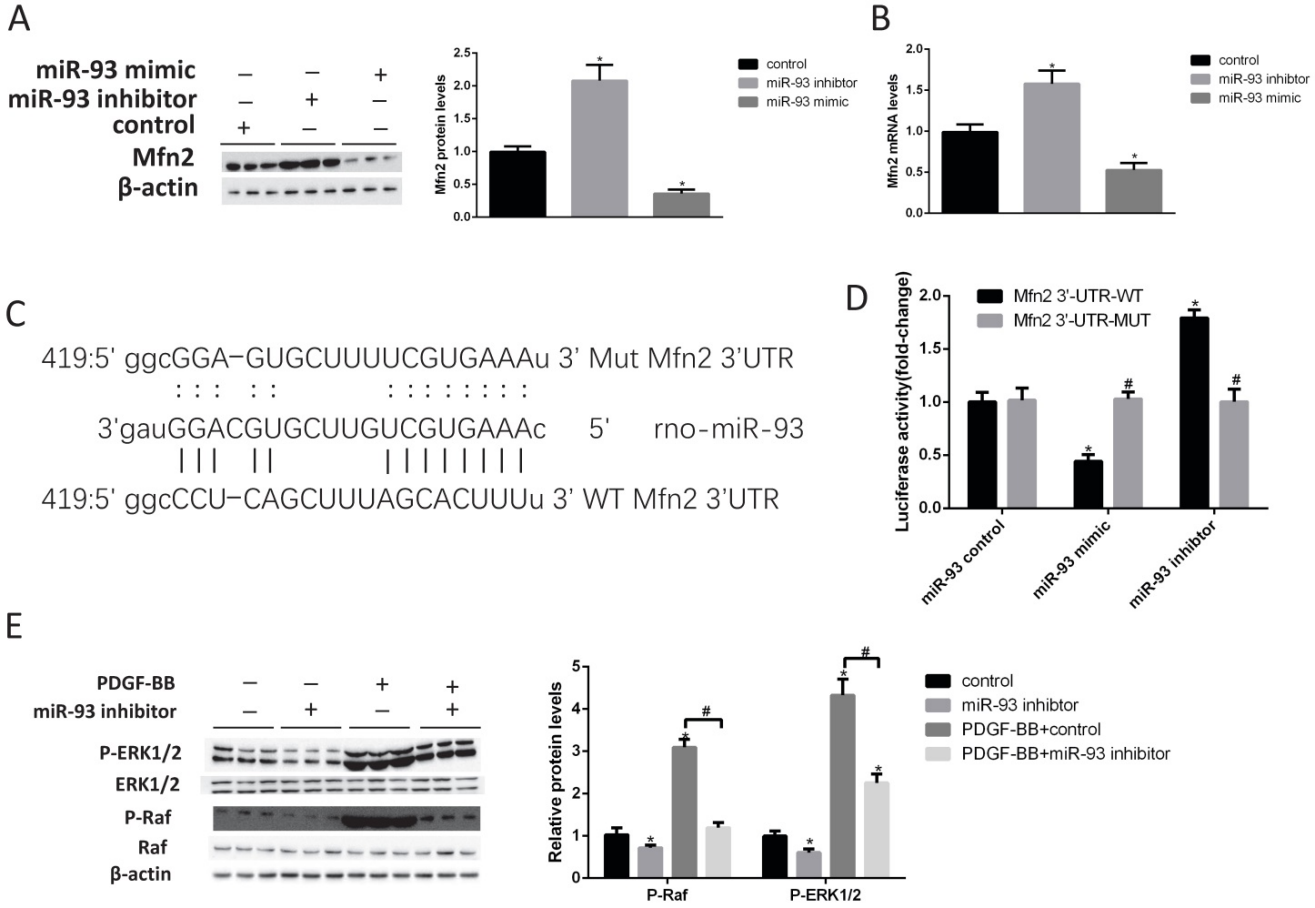
** Mfn2 is a target gene of miR-93.** (A) Western blot analysis of Mfn2 expression in VSMCs after transfection with either miR-93 mimic or miR-93 inhibitor (*P<0.05 versus control, n=4). (B) Effects of miR-93 mimic and inhibitor on Mfn2 mRNA levels determined by qRT-PCR (*P<0.05 versus control, n=4). (C) Schematic diagram illustrating the design of luciferase reporters with the wild-type Mfn2 3'UTR (WT Mfn2A 3'UTR) or the site-directed mutant Mfn2 3'UTR (MUT Mfn2 3'UTR). (D) HEK293 cells were co-transfected with miR-93 control, mimic or inhibitor with the WT or MUT 3'UTR of Mfn2, and luciferase activity was detected (*P<0.05 versus WT control, #P>0.05 versus MUT control, n=6). (E) VSMCs transfected with miR-93 control or miR-93 inhibitor were stimulated with or without PDGF-BB for 24 h and analyzed for P-ERK1/2 and P-Raf protein levels by western blot (*P<0.05 versus control, #P<0.05 compared between crossed lines, n=4).

To further validate the relationship between Mfn2 and miR-93, we treated rat VSMCs with miR-93 control, miR-93 mimic and miR-93 inhibitors. According to western blot (Fig. 5A) and qRT-PCR (Fig. 5B) results, the miR-93 mimic significantly decreased Mfn2 expression levels, whereas the miR-93 inhibitor increased Mfn2 expression level. The results showed that miR-93 and Mfn2 were negatively correlated. According to the miRBase analysis, Mfn2 has a miR-93 binding site in its 3'-UTR (Fig. 5C). To determine whether miR-93 binds directly to the 3'UTR of Mfn2 to affect its expression, the wild Mfn2 3'UTR (WT) and the mutated (MUT) Mfn2 3'UTR were reconstituted into the PsiCHECK-2 vector. The constructed vector was then co-transfected with the miR-93 control, mimic and inhibitor into HEK293 cells. As shown in Fig. 5D, the miR-93 mimic significantly inhibited the luciferase activity of the WT Mfn2 3'UTR, and the miR-93 inhibitor was significantly upregulated in the WT Mfn2 3'UTR. The MUT Mfn2 3'UTR, however, did not change significantly. Studies have shown that Mfn2 inhibits vascular smooth muscle proliferation by through inhibiting the Raf-ERK1/2 pathway 31. As shown in Fig. 5F-G, Raf and ERK1/2 phosphorylation levels were significantly decreased after transfection with the miR-93 inhibitor. The total Raf and ERK1/2 levels, however, were not changed. These results indicate that Mfn2 is a target gene for miR-93 and that miR-93 inhibits Mfn2 expression by binding to the 3'UTR of Mfn2. In addition, inhibiting miR-93 expression suppressed VSMC proliferation by depressing downregulating the Raf-ERK1/2 pathway.

### Role of Mfn2 in VSMC proliferation and migration

**Figure 6:**
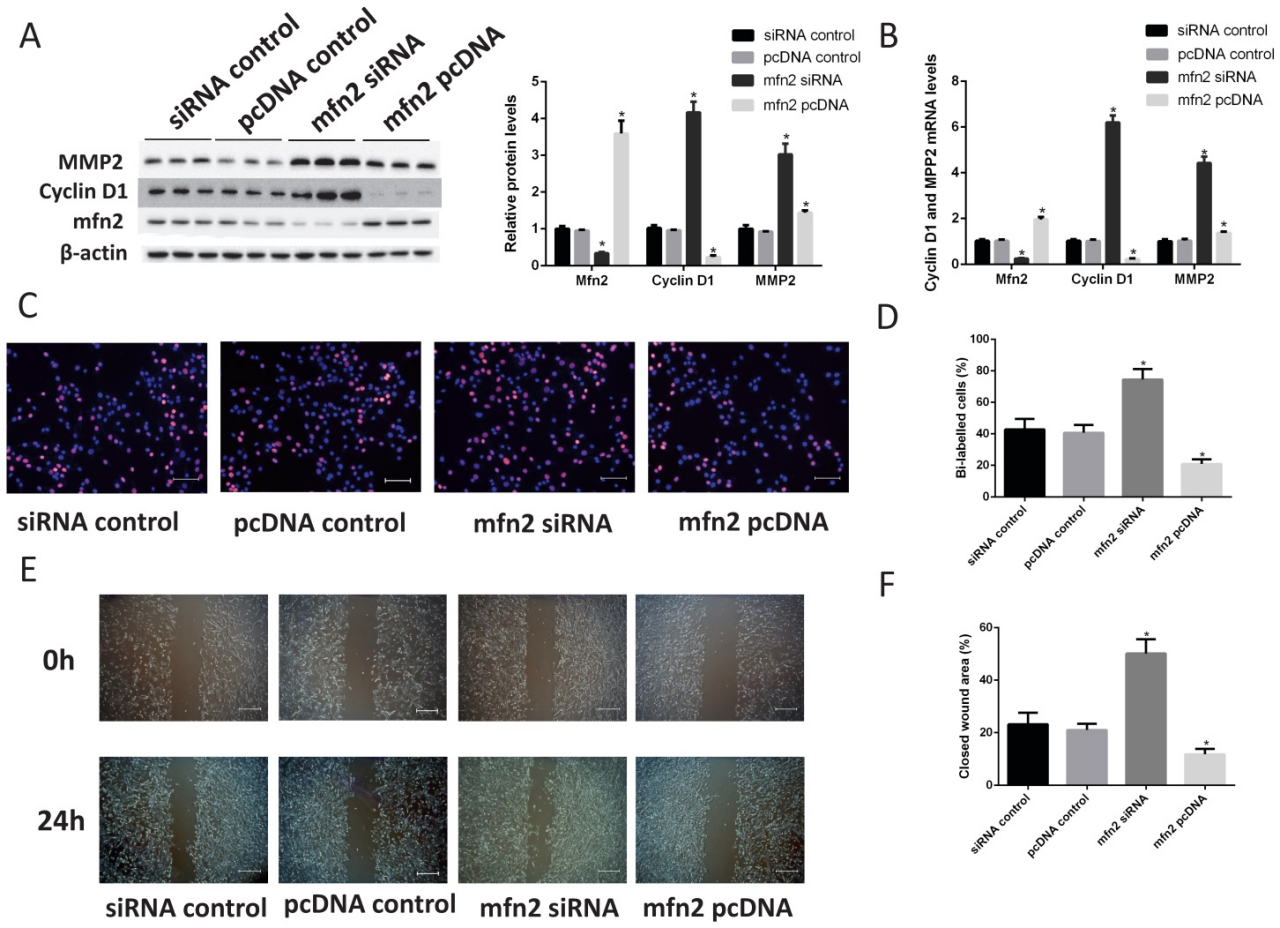
** Mfn2 inhibits VSMC proliferation and migration.** Rat VSMCs were transduced with Mfn2 siRNA or Mfn2 pcDNA. (A) Western blot analysis of Mfn2, cyclin D1 and MMP2 expression in VSMCs after transfection with either Mfn2 siRNA or Mfn2 pcDNA (*P<0.05 versus control respectively, n=4). (B) Analysis of Mfn2, cyclin D1 and MMP2 protein expression levels in rat VSMCs by qRT-PCR (*P<0.05 versus control respectively, n=4). (C) EdU incorporation in VSMCs; purple indicates the EdU-positive signal merged with nuclei stained with Hoechst 33342, and blue indicates nuclei. (D) EdU-positive cells were quantified by Image-Pro Plus (*P<0.05 versus control, n=4). (E) VSMC migration ability was measured by scratch wound assays. (F) Scratch wounds were quantified by using ImageJ to measure the closed wound area (*P<0.05 versus control, n=4).

According to a miRBase database search, Mfn2 is a potential miR-93 target because its 3'UTR is complementary to miR-93. Indeed, Mfn2 plays critical roles in VSMC proliferation and migration 34-35. To confirm the role of Mfn2 in VSMCs, we knocked down Mfn2 expression with siRNA and overexpressed Mfn2 with a Mfn2 pcDNA in VSMCs. According to western blot analyses, the protein levels of cyclin D1 and MMP2 were significantly upregulated when Mfn2 expression was knocked down. Furthermore, Mfn2 overexpression downregulated cyclin D1 and MMP2 (Fig. 6A). We also performed qRT-PCR assays to determine the mRNA levels of cyclin D1 and MPP2, and we obtained similar results (Fig. 6B). Next, we used EdU assays to confirm that Mfn2 overexpression can inhibit VSMC proliferation and that knocking down Mfn2 expression can promote proliferation (Fig. 6C-D). Furthermore, the closed wound area also confirmed that Mfn2 expression was negatively correlated with VSMC migration (Fig. 6E-F). These results demonstrated that Mfn2 can inhibit the proliferation and migration of VSMCs.

## Discussion

Vascular injury, remodeling and angiogenesis are the main causes of coronary heart disease and cerebrovascular disease. In response to injury and other stimuli, the expression levels of contractile proliferating proteins are decreased, and the expression levels of proliferating proteins are increased in VSMCs; these changes lead to increased cell proliferation, which is the primary pathogenesis of vascular restenosis after angioplasty 4, 5. Here, we identified miR-93 as a novel factor that modulates VSMC proliferation and migrations. First, miR-93 expression levels were increased in PDGF-BB-stimulated VSMCs in vitro and after balloon injury in vivo. Second, knocking down miR-93 inhibited PDGF-BB-induced VSMC proliferation and migrations. Third, knocking down miR-93 reduced the degree of carotid artery intimal hyperplasia induced by balloon injury. Fourth, Mfn2 was identified as a target gene of miR-93. Fifth, inhibiting miR-93 suppressed the Raf-ERK1/2 pathway, which plays an important role in the anti-proliferative effects of Mfn2 on VSMCs. Finally, to confirm the role of Mfn2 in VSMCs, we knocked down Mfn2 expression with siRNA and overexpressed Mfn2 with a Mfn2 plasmid in VSMCs. These results demonstrated that Mfn2 can inhibit the proliferation and migration of VSMCs. Taken together, these findings imply that the effect of miR-93 on restenosis could eventually be exploited therapeutically.

Previously, many studies have reported that microRNAs play very important roles in the proliferation and migration of VSMCs. For example, after PDGF-BB treatment, miR-21 expression is upregulated and promotes VSMC proliferations by inhibiting PTEN 32. Similarly, overexpression of miR-221/222 promotes proliferation of VSMCs by inhibiting P27KIP1/P57KIP1 16. However, due to the large number of miRNAs expressed in VSMCs, our understanding of miRNAs in VSMC regulation is not enough. Fortunately, evidence in this study demonstrates the important role of miR-93 in VSMCs. MiR-93 has been reported to promote the proliferation and migration of hepatocellular carcinoma, osteosarcoma cells and esophageal squamous cell carcinoma 20-22. However, the role of miR-93 in VSMCs has not been reported, and we have demonstrated that miR-93 expression levels are increased in injured arteries. Interestingly, a recent study used miRNA microarrays to show that miR-93 was upregulated after carotid artery injury 11. However, the mechanism of miR-93 regulation in vascular injury has not been explored. To further confirm these results in vitro, we treated VSMC primary cells with PDGF-BB to promote cell proliferation. Interestingly, miR-93 expression was dose- and time-dependently influenced by treating VSMCs with PDGF-BB. Furthermore, pathological observations in rats suggest that miR-93 inhibition can block neointimal hyperplasia induced by catheterization injury. In addition, VSMC proliferation and migrations were also suppressed by inhibiting miR-93 in vitro, and the expression levels of MMP2 and cyclin D1, which are critical regulators of VSMC proliferation and migration, were decreased 26-28. These results suggest that miR-93 promotes the proliferation and migration of VSMCs. Interestingly, our miR-93 inhibitor not only blocked VSMC proliferation induced by PDGF-BB,but also inhibited VSMC proliferation without PDGF-BB treatment. However, the miR-93 inhibitor bolcked only VSMC migration induced by PDGF-BB. This result implied that miR-93 regulated the proliferation and migration of VSMCs though different mechanisms.

Mfn2 plays a key role in regulating mitochondrial fusion and metabolic function 33. In addition, Mfn2 plays a pivotal role in T lymphocyte apoptosis through the mitochondrial death pathway 34. Chen Guanghui found that Mfn2 can significantly inhibit VSMC proliferation *in vitro* and *in vivo*, and this action has nothing to do with mitochondrial fusion; Furthermore rats and humans have 95.2% Mfn2 homology 35. A recent study indicated that miRNA can promote fibroblast differentiation by downregulating Mfn2 36. Jiang found that Mfn2 overexpression could inhibit VSMC migration to the intima and reverse the intimal thickening caused by intimal hyperplasia 34. Furthermore, Mfn2 has been reported to have an important effect on VSMCs and myocardial apoptosis 37, 38. In this study, we found that Mfn2 expression in VSMCs was downregulated by PDGF-BB treatment, and that miR-93 inhibition upregulated Mfn2 expression. Since miR-93 is upregulated after VSMCs are treated with PDGF-BB, we can say that the expression of miR-93 and Mfn2 are reversely-correlated. This finding was verified by transfecting a miR-93 mimic and miR-93 inhibitors into rat VSMCs. According to a miRBase prediction, Mfn2 is the target gene of miR-93. We used a luciferase reporter gene to confirm that miR-93 can bind to the 3'UTR region of Mfn2 and inhibit its fluorescence activity. These results demonstrated that Mfn2 is a target gene for miR-93.

Recent studies have shown that Mfn2 is an inhibitor of the proto-oncogene Ras; Mfn2 acts upon the myocardium and VSMCs by inhibiting the Raf-MAPK pathway to suppress cell proliferation, whereas the induction of apoptosis is mediated by inhibiting the Ras-PI3K- AKT pathway 30, 37-39. Jiang found that overexpression of Mfn2 overtly suppressed serum-evoked VSMC proliferation in culture, and blocked balloon injury induced neointimal VSMC proliferation and restenosis in rat carotid arteries. The Mfn2 antiproliferative effect was mediated by inhibition of ERK/MAPK signalling and subsequent cell-cycle arrest. They have also shown that Mfn2 markedly decreases serum-evoked activation of Raf and ERK1/2, and that the p21ras signature motif has an essential role in Mfn2-mediated inhibition of ERK1/2 signalling and growth arrest. These data strongly suggest that binding of Mfn2 to Ras causes a negative regulation of the Ras-Raf-MEK-ERK1/2 MAPK signalling pathway 34. We found that miR-93 inhibition could block the phosphorylation of Raf and ERK1/2. These results further confirmed that miR-93 regulates Mfn2 by regulating VSMC proliferation and migrations through the Raf-ERK1/2 pathway. Finally, we confirmed that Mfn2 can indeed inhibit the proliferation and migration of VSMCs by overexpressing and knocking down Mfn2 expression in VSMCs.

This is the first time that miR-93 has been shown to promote VSMC proliferation and migration. MiR-93 could also be used as a new target for treating the treatment of intracoronary stent restenosis. However, our study has limitations because miRNAs have multiple target genes. This fact does not exclude miR-93 from regulating VSMC proliferation and migration through other target genes. We have confirmed only that Mfn2 plays a role in this process, but the underlying mechanism regulating miR-93 expression is unclear. Consequently, we need to conduct further studies research to arrive at a conclusion.

## Figures and Tables

**Table 1:** Primers used for RT-PCR

MiR-93, Forward, 5'-ACACTCCAGCTGGGCAAAGTGCTGTTCGTGC-3' Reverse,5'- CTCAACTGGTGTCGTGGAGTCGGCAATTCAGTTGAGCTACCTGC-3'
Cyclin D1, Forward, 5'- TGCCACAGATGTGAAGTTCATT-3' Reverse,5'- GGAGGGAGTCCTTGTTTAGCC-3'
MMP2, Forward, 5'-TTTGGTCGATGGGAGCATGG-3' Reverse, 5'- ATAGCTGTGACCACCACCCT-3'
Mfn2, Forward,5'- GGACCTGAATCGGCACAGAG-3' Reverse,5'- GAGCAGGGACATCTCGTTTC-3'
GAPDH, Forward, 5'- -ATGACTCTACCCACGGCAAG-3' Reverse, 5'- TACTCAGCACCAGCATCACC-3'
U6, Sense primer, 5'-CTCGCTTCGGCAGCACA-3' Antisense primer, 5'-AACGCTTCACGAATTTGCGT-3'
